# Extracorporeal membrane oxygenation for severe acute respiratory distress syndrome in children with leukemia/lymphoma: A retrospective case series

**DOI:** 10.3389/fped.2022.955317

**Published:** 2022-09-08

**Authors:** Yucai Zhang, Yiping Zhou, Jingyi Shi, Yijun Shan, Ting Sun, Chunxia Wang, Jingbo Shao, Yun Cui

**Affiliations:** ^1^Department of Critical Care Medicine, Shanghai Children’s Hospital, Shanghai Jiao Tong University School of Medicine, Shanghai, China; ^2^Pediatric Extracorporeal Life Support Center, Institute of Pediatric Infection, Immunity, and Critical Care Medicine, Shanghai Jiao Tong University School of Medicine, Shanghai, China; ^3^Institute of Pediatric Critical Care, Shanghai Jiao Tong University, Shanghai, China; ^4^Department of Hematology and Oncology, Shanghai Children’s Hospital, Shanghai Jiao Tong University School of Medicine, Shanghai, China

**Keywords:** acute respiratory distress syndrome (ARDS), extracorporeal membrane oxygenation, leukemia, lymphoma, child

## Abstract

**Objective:**

The cancer patients with severe acute respiratory distress syndrome (ARDS) benefit from extracorporeal membrane oxygenation (ECMO) remains unanswered. We analyzed clinical characteristics and outcomes of pediatric patients with leukemia/lymphoma who developed ARDS and treated with ECMO.

**Methods:**

Pediatric leukemia or lymphoma patients with ARDS who underwent ECMO between August 2017 and December 2021 were retrospectively analyzed in a tertiary pediatric intensive care unit (PICU).

**Results:**

Seven patients with median age 53 (IQR 42–117) months and 4 males were included. Six cases of leukemia [5 of acute lymphocytic leukemia (ALL) and 1 of acute myelogenous leukemia (AML, M5)] and 1 of non-Hodgkin lymphoma with severe ARDS received ECMO on chemotherapy period. The etiology of ARDS is community or chemotherapy-associated bacterial or/and fungal or viral infection. All the patients received chemotherapy in the 2 weeks prior to ECMO and five were neutropenic at initial ECMO. Six cases underwent veno-arterial ECMO (VA ECMO) and 1 for veno-venous ECMO (VV-ECMO). The median duration of ECMO support was 122 (IQR 56–166) hours. Overall, 42.9% (three of seven) survived to hospital discharge and 6 months survival rate was 28.6% (two of seven). Bleeding was the main ECMO-associated complication occurring in 7 patients, followed by nosocomial infection in 4 cases. All the patients required vasopressor support, and 6 received continuous renal replacement therapy (CRRT).

**Conclusion:**

Our experiences suggest that rescue ECMO provides a selective treatment strategy in childhood hematologic malignancies with severe ARDS.

## Introduction

Pediatric acute respiratory distress syndrome (pARDS) remains with high mortality according to an international, prospective, cross-sectional, observational study on 145 pediatric intensive care units (PICUs) from 27 countries (1). Patients with cancer are at increased risk for development of acute respiratory distress syndrome (ARDS), either due to underlying malignancy or due to treatment-related complication. Despite improvements made in recent years, the mortality attributable to ARDS remains high in this population (2, 3). Extracorporeal membrane oxygenation (ECMO) is a treatment option for patients with severe ARDS in whom conventional mechanical ventilation fails (4–7). Recently data from the registry of the Extracorporeal Life Support Organization (ELSO) indicated a hospital discharge rate of 58% in adults and 69% in pediatric pulmonary support with ECMO (8). However, due to worse outcomes with malignancy, eligibility of patients with cancer for ECMO support is an ongoing controversy.

Unsurprisingly, the confrontation of with critically ill patients with cancer is associated with significantly increased mortality risk. With regard to the severity of underlying malignancy, the prognosis of cancer patients is uncertainty and at high risk of life support-related adverse events, the challenge remains in times for the use of ECMO. Several studies showed that the value of veno-venous ECMO (VV-ECMO) was still unsatisfactory in adult patients with cancer with ARDS (9–11). Kochanek et al. (10) reported the sadly 60-day overall survival rate of patients with cancer who required VV-ECMO in 19 German and Austrian hospitals. However, there is only limited information available on pARDS with cancer or leukemia that received ECMO support. Therefore, we conducted a retrospective case series analysis to describe clinical characteristics and outcomes of patients with leukemia/lymphomas with pARDS who received ECMO.

## Materials and methods

We performed an analysis of children with hematologic malignancies who developed ARDS requiring rescue therapy with ECMO at our PICU from August 2017 to November 2021. At baseline of cytogenetics, the etiology of ARDS and reasons of PICU admission were recorded. Markers such as PaO_2_/FiO_2_ ratio, PaCO_2_, and lactate were recorded at initiation of ECMO. Mainly, laboratory variables including platelet count, coagulation function, and renal and liver functions were recorded. ECMO modality, ventilator parameters, vasopressor therapies, vasoactive inotropic score (VIS), and need for continuous renal replacement therapy (CRRT) were analyzed.

Before and after receiving ECMO support, the lung protective ventilation strategy is performed in our PICU. The ventilation strategy on ECMO support is goal of positive end-expiratory pressure (PEEP) of 10–12 cm H_2_O, inspiratory pressures less than 25 cm H_2_O, and a fraction of inhaled oxygen (FiO_2_) 0.4–0.5. Tidal volume goal is maintained at less 3–5 ml/kg ideal body weight until pulmonary recovery. Unfractionated heparin infusion was titrated to activated coagulation time (ACT) level of 150–180 min so long as activated partial thromboplastin time (aPTT) remained less than 80 s during ECMO. The patients were infused with exogenous platelets or prothrombin complexes, or fresh frozen plasma for maintaining a target platelet count of at least 25 × 10^9^/L and a fibrinogen level of 1.2 g/L in severe thrombocytopenia or hypofibrinogenemia. Weaning from ECMO is performed according to Pediatric Acute Lung Injury Consensus Conference (PALICC) guidelines (12). Our ECMO center is one of major pediatric ECMO centers in China and a registered ELSO member (No. 663).

This retrospective analysis was approved by the ethics committee of Shanghai Children’s Hospital, Shanghai Jiao Tong University School of Medicine (Protocol: 2020R063-E01). Because no additional interventions were performed, informed consent was waived by the institutional review board. After removing the patient privacy information, all the data used were anonymized and could not be traced back to individual patients.

## Results

A total of 7 children with hematologic malignancies developed severe ARDS requiring ECMO support. Four patients were males and 3 were females with median age of 53 months (IQR 42–117). Five cases were acute lymphocytic leukemia (ALL), 1 was acute myeloid leukemia (AML, M5, case2), and 1 was non-Hodgkin lymphoma (case 3). The main comorbidities and clinical characteristics for each patient are outlined in Table 1. The etiology of ARDS is community or chemotherapy-associated bacterial or/and fungal or viral infections. All the patients received chemotherapy in the 2 weeks prior to ECMO, and five were neutropenic at initial ECMO. Six patients received CRRT because of acute kidney injury (AKI) and/or fluid overload. Three cases were treated with 1–4 times of therapeutic plasm exchanges (TPEs) for liver dysfunction. All the patients were on broad-spectrum antibiotics and antifungal prophylaxis/treatment (Table 2).

**TABLE 1:** Baseline characteristics of patients at initial ECMO support.

Variable	Case 1	Case 2	Case 3	Case 4	Case 5	Case 6	Case 7
**Etiology for ARDS**	Elizabethkingia meningoseptica	Gram-negative bacillus	Klebsiella pneumoniae	Streptococcus pneumoniae + aspergillus flavus + RSV	Bordetella pertussis + candida albicans	Klebsiella pneumoniae	Streptococcus pneumoniae + influenza virus B
**Comorbidities**							
Shock	Yes	Yes	Yes	Yes	Yes	Yes	Yes
AKI	No	No	Yes	Yes	Yes	Yes	No
Liver dysfunction	Yes	Yes	Yes	Yes	Yes	Yes	No
Coagulopathy	Yes	Yes	Yes	Yes	Yes	Yes	Yes
Fluid overload	>5%	<5%	>10%	>10%	>5%	<5%	>10%
MODS	Yes	Yes	Yes	Yes	Yes	Yes	Yes
**MV parameter before ECMO**							
p-plat, cmH_2_O	31	29	31	30	31	34	33
Peak pressure, cmH_2_O	34	30	35	34	35	39	38
PEEP, cmH_2_O	13	8	16	14	12	18	15
**PICU days of ECMO cannulation**	1	4	1	7	9	1	3
**MV days of ECMO cannulation**	2	5	1	4	6	1	3
**ECMO modality**	V-A	V-A	V-A	V-V	V-A	V-A	V-A
**Anticoagulation**	Heparin	Heparin	Heparin	Heparin	Heparin	Heparin	Heparin

ARDS, acute respiratory distress syndrome; ECMO, extracorporeal membrane oxygenation; AML, acute myelogenous leukemia; ALL, acute lymphocytic leukemia; AKI, acute kidney injury; MODS, multiple organ dysfunction syndrome; MV, mechanical ventilation; PEEP, positive end-expiratory pressure; PICU, pediatric intensive care unit; RSV, respiratory syncytial virus; p-plat, pressure of plat.

**TABLE 2:** Clinical and laboratory characteristics in pARDS on ECMO with leukemia/lymphoma.

Variable	Case 1	Case 2	Case 3	Case 4	Case 5	Case 6	Case 7
**Values at initial ECMO**							
MAP, mmHg	57	53	30	64	56	60	64
CI, L/min/m^2^	3.0	2.7	2.4	2.6	3.4	2.7	2.9
PaO_2_/FiO_2_, mmHg	46	55	79	46	64	45	63
PaCO_2_, mmHg	58	59	48	79	61	60	92
lactate, mmol/L	3.3	3.1	6.2	1.6	8.6	2.9	2.4
Platelets, 10^9^/L	105	27	5	44	105	16	79
INR	1.12	1.38	1.66	1.07	2	1.29	2.29
Fibrinogen, g/L	3.1	0.66	1.92	2.12	2.81	2.45	3.36
D-dimer	2.23	19.02	3.23	15.5	19.14	4.15	1.85
VIS	100	200	200	120	150	150	100
**Peak/nadir values**							
Leucocytes peak/nadir, 10^9^/L	25.4/6.4	0.14/0.01	0.96/0.02	34.5/0.2	54.2/18.3	0.3/0.1	25.5/3.5
Platelets nadir, 10^9^/L	27	2	5	4	41	10	31
Number days with platelets < 50 × 10^9^/L	4	13	2	4	2	3	2
Peak INR	1.85	1.61	5.98	1.48	2.87	2.17	3.97
Fibrinogen nadir, g/L	0.67	0.58	0.41	1.06	1.12	0.51	1.41
Peak D-dimer, mg/L	66.15	22.01	3.91	29.57	25.04	20.01	26.08
Peak CRP, mg/L	225	273	203	219	210	163	136
Peak procalcitonin, mg/L	8.66	12.61	9.98	26.64	25.97	98.17	8.06
Albumin nadir, g/L	29.5	21.9	21.0	33.3	28.5	26.4	33.0
Peak bilirubin, μmol/L	106.5	329.6	51.4	470.6	184.3	131.4	14.8
Peak ALT, U/L	1,308	18	816	91	357	49	14
Peak creatinine, mmol/L	49	55	83	95	168	75	66
Peak BUN, mmol/L	15.8	33.7	10.3	25.8	24.4	15.5	7.3
**Vasoactive inotropic drugs on ECMO, peak**							
Norepinephrine, μg/kg.h	0.2	2	2	0.1	2	2	0.1
Epinephrine, μg/kg.h	0.1	2	2	/	0.5	2	/
Dopamine, μg/kg.h	10	15	10	10	20	20	/
Dobutamine, μg/kg.h	10	15	10	5	15	10	/
**Peak VIS on ECMO**	20	400	400	20	250	400	20
**Transfusions on ECMO**							
pRBC, ml/kg	77	200	30	123	29	33	83
FFP, ml/L	36	53	67	88	35	32	69
Platelets, U	10	50	30	60	10	30	20
**ECMO-associated complications**							
Bleeding/hemorrhage	GI and catheter site	GI and catheter site	Diffuse alveolar	Pleural cavity and catheter site	GI	GI and catheter site	Intracranial
Nosocomial infection	Lung and catheter-related/Ab	Bloodstream/ enterobacter cloacae	No	Lung/Ab	Bloodstream/Ab	No	No
**Duration of ECMO, hours**	223	161	49	166	56	82	122
**Prone ECMO/MV**	Yes	Yes	No	Yes	Yes	No	Yes
**Duration of MV, days**	12	11	2	19	10	2	7
**CRRT, hours**	61	0	19	273	42	20	117
**TPE, numbers**	0	3	0	4	1	0	0
**Outcome**	Survival	Died	Died	Survival	Died	Died	Survival
**Reasons of death**	/	ARF	Alveolar hemorrhage	/	MODS	Shock	/

MAP, mean arterial pressure; CI, Cardiac index; BUN, blood urea nitrogen; ECMO, extracorporeal membrane oxygenation; CRP, C reactive protein; GI, Gastro-intestine; CRRT, continuous renal replacement therapy; pRBCs, packed red blood cells; FFP, fresh frozen plasma; Ab, Acinetobacter baumannii; TPE, therapeutic plasm exchange; ARF, acute respiratory failure; MV, mechanical ventilation.

The median duration of mechanical ventilation before ECMO support was 3 days (range 1–6 days). The median duration of ECMO support was 122 h (IQR 56–166). All the patients required a high dosage of vasopressor support (VIS ≥ 100). Six patients received veno-arterial ECMO (VA-ECMO) and 1 received VV-ECMO. Among six of the VA ECMO patients, four underwent surgical cut-down cannulation performed *via* neck vessels (right internal jugular vein-right carotid artery), and two patients underwent percutaneous femoral cannulation (femoral vein-femoral artery). One VV ECMO patient underwent percutaneous cannulation *via* the right internal jugular vein-femoral vein. After the initiation of ECMO, the vasoactive drugs were gradually downregulated, and the parameters of ventilator rapidly were reduced to protective ventilation in the survivors.

## Outcome

Three (3/7) patients survived and was discharged from the hospital. Among of 3 survivors, one patient recovered from ALL with severe ARDS received ECMO and healthy hospital discharged, but died 5 months later because of new septic shock. During the ECMO support, all patients were complicated with bleeding and 1 case with intracranial hemorrhage. One patient died of diffuse alveolar hemorrhage at 49 h of ECMO support. The causes of other non-survivors are outlined in Table 2. Moreover, the nosocomial infection on ECMO was complicated in 2 non-survivors and 2 in hospital survivor (Table 2).

## Discussion

ECMO support in patients with hematologic malignancies who develop severe ARDS remains controversial. In our study, the overall survival rate was 42.9% (three of seven) for hospital discharge and 28.6% (two of seven) for 6-month survival follow-up. The outcome of pediatric patients with leukemia/lymphoma with severe ARDS receiving ECMO as a rescue treatment was still unsatisfactory.

The mortality for patients with severe pARDS was about 33% (95% CI: 26–41) in the pARDS incidence and epidemiology (PARDIE) study (1). ECMO is a rescue treatment for patients with severe ARDS (8). However, the benefits of ECMO for specific subgroups of patients remain uncertain and a matter of debate (9, 13, 14). A few studies have been published on outcomes of ECMO in patients with malignant diseases. According to recent studies, the median overall survival rate was still low in adult patients with hematologic malignancies who developed ARDS receiving ECMO (9, 10, 15). More recently, the largest study reported the outcome of 297 adult patients with cancer with severe ARDS, and the 60-day survival rate was 26.8% (95% CI 22.1–32.4%). After propensity score matching, there was no significant difference in the survival of patients receiving ECMO and those managed with mechanical ventilation only (*p* = 0.089) (10). Only few cases reported the role of ECMO in pediatric patients with patient (16, 17). The larger pediatric case series study on this topic is that Cortina et al. (16) reported 9 patients of childhood leukemia with ARDS receiving ECMO, 5 (56%) patients survived to ECMO decannulationand4 (44%) survived to hospital discharge. In our case series, 4 (42.9%) of 7 patients survived and were discharged from the hospital, but 1 died 5 months later. This result suggested that rescue ECMO may be offered to selected children with leukemia/lymphoma-complicated ARDS.

The main factors affecting the success rate of ECMO include the timing of intervention, difficulty in primary disease treatment, and severity of ECMO-associated complications. The most frequent complications during ECMO are bleeding and ECMO-related infections. Higher risk for infectious and bleeding complications were main causes of lower success rates in patients with malignancy (18–20). Platelet count had been regarded as an indicator of the general severity of illness and a risk factor for bleeding-related morbidity and mortality. Kochanek et al. (10) found that low platelet count (95% CI.996–0.999; *p* = 0.0001, per 1,000 platelets/μl), elevated lactate levels (95% CI 1.012–1.084; *p* = 0.0077), and progressive disease (95% CI 1.081–3.238; *p* = 0.0253) were independent adverse prognostic factors for overall survival. We observed bleeding complication in all the patients, and two of the children had severe bleeding episodes (severe alveolar bleeding and intracranial hemorrhage). We infused fresh frozen plasma, prothrombin complexes, and exogenous platelets to guarantee the target platelets count > 25 × 10^9^/L and a fibrinogen level of 1.2 g/L in severe thrombocytopenia or hypofibrinogenemia. Only one case had disastrous alveolar hemorrhage who died at 36 h of ECMO support.

To improve the outcome of patients with hematologic malignancies with ARDS, it will be important to better understand the characteristics for ECMO modality and the timing. In severe pARDS, VV-ECMO patients displayed a significantly lower in-hospital mortality (50% vs. 26.9%, *p* = 0.044) compared to VA-ECMO in a prospective multicenter study (5). According to the PALICC (12) suggestion, there is a trend toward a preference for VV- ECMO for respiratory support in children. However, the choice of whether to use VV or VA- ECMO must be based on the assessment of the individual child, in particular whether or not there is circulatory compromise. In the absence of cardiac or circulatory dysfunction, expert opinion favors the choice of VV- ECMO. In the present study, because of hemodynamic instability with higher dosage of vasopressors (VIS ≥ 100), only 1 patient received VV-ECMO and 6 cases received VA-ECMO. Although the vasopressor dose was gradually withdrawn after initiating ECMO in the survivors, we cannot determine whether the poor discharge survival rate was associated with ECMO modality and hemodynamic disorders.

We did not routinely use high-frequency ventilation to rescue the severe ARDS prior to ECMO. High-frequency oscillatory (HFO) ventilation is not recommended according to “an expert opinion” (21). HFO is theoretically an attractive technique that could theoretically ensure adequate gas exchange and avoid excessive tidal stretching and atelectrauma. However, whether this technique could be used in very severe PARDS before ECMO salvage therapy warrants further investigation.

## Conclusion

Our case series is limited data by the small sample and by the retrospective analysis. However, it indicates that ECMO may provide a selective treatment for childhood hematologic malignancies with severe ARDS.

## Data availability statement

The raw data supporting the conclusions of this article will be made available by the authors, without undue reservation.

## Ethics statement

This study was approved by the Ethics Committee of Shanghai Children’s Hospital, Shanghai Jiao Tong University School of Medicine, China (Approval Number: 2016R011-E01). Written informed consent was waived by the institutional review board.

## Author contributions

YCZ and YPZ conceived the study and collected the data. JYS, YS, TS, and JBS collected the data. YCZ, YPZ, and YC wrote the first draft of the manuscript. CW provided comments and approved the final version of the manuscript. YC and JBS were responsible for the design of the study. All authors critically reviewed and edited the manuscript and read and approved the final version.

## Abbreviations

PICU: pediatric intensive care unit
ECMO: extracorporeal membrane oxygenation
ELSO: Extracorporeal Life Support Organization
VA-ECMO: venoarterial extracorporeal membrane oxygenation
VV-ECMO: venovenous extracorporeal membrane oxygenation
pARDS: pediatric acute respiratory distress syndrome
ALL: acute lymphocytic leukemia
AML: acute myelogenous leukemia
CRRT: continuous renal replacement therapy/renal replacement therapy
VIS: vasoactive inotropic score
PEEP: positive end-expiratory pressure
FiO_2_: fraction of inhaled oxygen
ACT: activated coagulation time
aPTT: activated partial thromboplastin time
AKI: acute kidney injury
TPE: therapeutic plasm exchange
PARDIE: pediatric acute respiratory distress syndrome incidence and epidemiology
MODS: multiple organ dysfunction syndrome
MV: mechanical ventilation
RSV: respiratory syncytial virus
p-plat: pressure of plat
MAP: mean arterial pressure
CI: cardiac index
CRP: C reactive protein
GI: gastro-intestine
pRBCs: packed red blood cells
FFP: fresh frozen plasma
Ab: acinetobacterbaumannii
ARF: acute respiratory failure.

## References

[1] Khemani RG Smith L Lopez-Fernandez YM Kwok J Morzov R Klein MJ Paediatric acute respiratory distress syndrome incidence and epidemiology (PARDIE): an international, observational study. *Lancet Respir Med.* (2019) 7:115–28. 10.1016/S2213-2600(18)30344-8 30361119 PMC7045907

[2] Prasertsan P Anuntaseree W Ruangnapa K Saelim K Geater A. Severity and mortality predictors of pediatric acute respiratory distress syndrome according to the pediatric acute lung injury consensus conference definition. *Pediatr Crit Care Med.* (2019) 20:e464–72. 10.1097/PCC.0000000000002055 31274780

[3] Yehya N Harhay MO Klein MJ Shein SL Piñeres-Olave BE Izquierdo L Predicting mortality in children with pediatric acute respiratory distress syndrome: a pediatric acute respiratory distress syndrome incidence and epidemiology study. *Crit Care Med.* (2020) 48:e514–22. 10.1097/CCM.0000000000004345 32271186 PMC7237024

[4] Combes A Schmidt M Hodgson CL Fan E Ferguson ND Fraser JF Extracorporeal life support for adults with acute respiratory distress syndrome. *Intensive Care Med.* (2020) 46:2464–76. 10.1007/s00134-020-06290-1 33140180 PMC7605473

[5] Cui Y Zhang Y Dou J Shi J Zhao Z Zhang Z Venovenous vs. venoarterial extracorporeal membrane oxygenation in infection-associated severe pediatric acute respiratory distress syndrome: a prospective multicenter cohort study. *Front Pediatr.* (2022) 10:832776. 10.3389/fped.2022.832776 35391748 PMC8982932

[6] Thompson K Staffa SJ Nasr VG Zalieckas JM Fynn-Thompson F Boyer D Mortality after lung transplantation for children bridged with extracorporeal membrane oxygenation. *Ann Am ThoracSoc.* (2022) 19:415–23. 10.1513/AnnalsATS.202103-250OC 34619069

[7] Combes A Peek GJ Hajage D Hardy P Abrams D Schmidt M. ECMO for severe ARDS: systematic review and individual patient data meta-analysis. *Intensive Care Med.* (2020) 46:2048–57. 10.1007/s00134-020-06248-3 33021684 PMC7537368

[8] Extracorporeal Life Support Organization Ecls Registry Report, (2022). Available. online at: https://www.elso.org/Portals/0/Files/Reports/2022_April/International%20Report%20April%202022.pdf (accessed May 26, 2022).

[9] Schmidt M Schellongowski P Patroniti N Taccone FS Reis Miranda D Reuter J Six-month outcome of immunocompromised severe ARDS patients rescued by ECMO. An international multicenter retrospective study. *Am J RespirCrit Care Med.* (2018) 197:1297–1297. 10.1164/rccm.201708-1761OC 29298095 PMC7252852

[10] Kochanek M Kochanek J Böll B Eichenauer DA Beutel G Bracht H. Veno-venous extracorporeal membrane oxygenation (vv-ECMO) for severe respiratory failure in adult cancer patients: a retrospective multicenter analysis. *Intensive Care Med.* (2022) 48:332–42. 10.1007/s00134-022-06635-y 35146534 PMC8866383

[11] Schmidt M Combes A Shekar K. ECMO for immunosuppressed patients with acute respiratory distress syndrome: drawing a line in the sand. *Intensive Care Med.* (2019) 45:1140–2. 10.1007/s00134-019-05632-y 31087113

[12] Dalton HJ Macrae DJ Pediatric acute lung injury consensus conference group. Extracorporeal support in children with pediatric acute respiratory distress syndrome: proceedings from the pediatric acute lung injury consensus conference. *Pediatr Crit Care Med.* (2015) 16:S111–7. 10.1097/PCC.0000000000000439 26035361

[13] Combes A Hajage D Capellier G Demoule A Lavoué S Guervilly C Extracorporeal membrane oxygenation for severe acute respiratory distress syndrome. *N Engl J Med.* (2018) 378:1965–75. 10.1056/NEJMoa1800385 29791822

[14] Barbaro RP Xu Y Borasino S Truemper EJ Watson RS Thiagarajan RR Does extracorporeal membrane oxygenation improve survival in pediatric acute respiratory failure? *Am J RespirCrit Care Med.* (2018) 197:1177–86. 10.1164/rccm.201709-1893OC 29373797 PMC6019927

[15] Huprikar NA Peterson MR DellaVolpe JD Sams VG Lantry JH Walter RJ Salvage extracorporeal membrane oxygenation in induction-associated acute respiratory distress syndrome in acute leukemia patients: a case series. *Int J Artif Organs.* (2019) 42:49–54. 10.1177/0391398818799160 30223700

[16] Cortina G Neu N Kropshofer G Meister B Klingkowski U Crazzolara R. Extracorporeal membrane oxygenation offers long-term survival in childhood leukemia and acute respiratory failure. *Crit Care.* (2018) 22:222. 10.1186/s13054-018-2134-6 30241492 PMC6151048

[17] Kebudi R OflazSozmen B Bahar M Paker T Hacı I Ekinci A Prolonged extracorporeal membrane oxygenation in pediatric leukemia with severe acute respiratory distress syndrome and persistent fungemia. *Pediatr Blood Cancer.* (2021) 68:e28966. 10.1002/pbc.28966 33629444

[18] Gow KW Heiss KF Wulkan ML Katzenstein HM Rosenberg ES Heard ML Extracorporeal life support for support of children with malignancy and respiratory or cardiac failure: the extracorporeal life support experience. *Crit Care Med.* (2009) 37:1308–16. 10.1097/CCM.0b013e31819cf01a 19242331

[19] Smith S Butt W Best D MacLaren G. Long-term survival after extracorporeal life support in children with neutropenic sepsis. *Intensive Care Med.* (2016) 42:942–53. 10.1007/s00134-015-4163-9 26650054

[20] Di Nardo M Locatelli F Palmer K Amodeo A Lorusso R Belliato M Extracorporeal membrane oxygenation in pediatric recipients of hematopoietic stem cell transplantation: an updated analysis of the extracorporeal life support organization experience. *Intensive Care Med.* (2014) 40:754–6. 10.1007/s00134-014-3240-9 24556913

[21] Chiumello D Brochard L Marini JJ Slutsky AS Mancebo J Ranieri VM Respiratory support in patients with acute respiratory distress syndrome: an expert opinion. *Crit Care.* (2017) 21:240. 10.1186/s13054-017-1820-0 28899408 PMC5596474

